# Lifestyle and occupational risks assessment of bladder cancer using machine learning‐based prediction models

**DOI:** 10.1002/cnr2.1860

**Published:** 2023-07-05

**Authors:** Naser Shakhssalim, Atefeh Talebi, Mohammad‐Taha Pahlevan‐Fallahy, Kasra Sotoodeh, Hamid Alavimajd, Nasrin Borumandnia, Maryam Taheri

**Affiliations:** ^1^ Urology and Nephrology Research Center Shahid Beheshti University of Medical Sciences Tehran Iran; ^2^ British Heart Foundation Cardiovascular Research Centre University of Glasgow Glasgow UK; ^3^ Students' Scientific Research Center, School of Medicine Tehran University of Medical Sciences Tehran Iran; ^4^ Department of Biostatistics, School of Allied Medical Sciences Shahid Beheshti University of Medical Sciences Tehran Iran

**Keywords:** bladder cancer, machine learning, predictive models

## Abstract

**Background:**

Bladder cancer, one of the most prevalent cancers globally, can be regarded as considerable morbidity and mortality for patients. The bladder is an organ that comes in constant exposure to the environment and other risk factors such as inflammation.

**Aims:**

In the current study, we used machine learning (ML) methods and developed risk prediction models for bladder cancer.

**Methods:**

This population‐based case–control study is focused on 692 cases of bladder cancer and 692 healthy people. The ML, including Neural Network (NN), Random Forest (RF), Decision Tree (DT), Naive Bayes (NB), Gradient Boosting (GB), and Logistic Regression (LR), were applied, and the model performance was evaluated.

**Results:**

The RF (AUC = .86, precision = 79%) had the best performance, and the RT (AUC = .78, precision = 73%) was in the next rank. Based on variable importance analysis in RF, recurrent infection, bladder stone history, neurogenic bladder, smoking and opium use, chronic renal failure, spinal cord paralysis, analgesic, family history of bladder cancer, diabetic mellitus, low dietary intake of fruit and vegetable, high dietary intake of ham, sausage, can and pickles were respectively the most important factors, which effect on the probability of bladder cancer.

**Conclusion:**

Machine learning approaches can predict the probability of bladder cancer according to medical history, occupational risk factors, and dietary and demographical characteristics.

## 1 INTRODUCTION

Cancer is one of the main causes of death and morbidity nowadays. According to the global cancer observatory, bladder cancer is the 10th most frequent cancer in the general population.^1^ Worldwide studies of cancer demonstrate that 1 out of every 100 men or 400 woman experience bladder cancer during their lifetime.^2^ Many risk factors contribute to bladder cancer, which can be categorized into genetic predisposition and exposure to external carcinogens. Evidence showed that many cases of bladder cancer could be attributed to external risk factors, namely smoking and tobacco, family history of smoking or tobacco use or workplace exposure to cigarette smoke, and past medical history such as a history of bladder stones, neurogenic bladder, recurrent urinary tract infections (UTI), family history of bladder cancer, and diabetes mellitus.^3^, ^4^, ^5^ Exposure to certain materials like petroleum and its derivatives, paint, some herbal drugs, and excessive use of analgesics have also been regarded as risk factors in some investigations.^6^ Also, some studies have suggested that the diet and the type of foods consumed by a person might be useful in predicting the risk of bladder cancer.^7^

Machine learning (ML) approaches is a branch of computer science in medical research. Being tremendously on the rise, many researchers apply different ML methods to develop models to predict the risk of diseases, make diagnostic criteria more accurate, or even diagnose the outcome of treatment based on different factors. Knowing the risk factors might help to strengthen the primary prevention plans of healthcare systems to achieve their goals and make them more accurate. The goal of the study, which included a relatively large sample size population, was to employ ML strategies to determine the influence each risk factor has on bladder cancer.

## 2 METHODS

This population‐based case–control study includes 692 cases of bladder cancer and 692 healthy people. Bladder cancer patients were selected from the cancer registry system, and one of the right door neighbors in each case, matched based on sex and age, was recruited as a control. More details are presented in a previously published article.^8^ Bladder cancer status was considered the dependent variable, and medical history, family history of bladder cancer, occupational risk factors, dietary and lifestyle, and demographical characteristics were the independent factors. Medical history of diabetes mellitus, chronic renal failure (CRF), bladder stone, neurogenic bladder, spinal paralysis, and recurrent UTI was included. Occupational risk factors, such as exposure to petroleum, paint, and its derivatives, leather, weavers, and spinners, were regarded. In addition, the amount of sausage, ham, and canned food usage per week and fruits, vegetables, and pickles per day, were the dietary factors entered in the ML model. Finally, lifestyle factors, which can affect bladder cancer, were considered. Those include a history of smoking, excessive analgesic use, opium and herbal medications, and hair coloring. The study protocol was approved by the Ethics Committee of the Urology and Nephrology Research Center at Shahid Beheshti University of Medical Sciences with registration number 221 and Ethics Code 860328/39. All methods were performed in accordance with the Declaration of Helsinki.

### 2.1 Data preprocessing and model development

The statistically missing values were synthesized in the data preprocessing phase. The large dataset includes 1384 samples, and 27 features and bladder status were used as response variables. The binning discretization mechanisms were carried out to convert features with more discrete values into categorical values. Moreover, normalization, centering to the mean and scaling to a standard deviation of 1, was served in continuous variables. Due to the equal sample of health and patients' number, the distribution was considered balanced.

**FIGURE 1:**
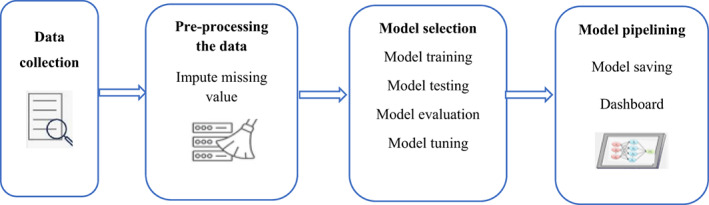
Framework of methodology.

In the study, Neural Network (NN), Random Forest (RF), Decision Tree (DT), Naive Bayes (NB), Gradient Boosting (GB), and Logistic Regression (LR) were developed. The multi‐layer perceptron network was applied for NN modeling. The activation function of the rectified linear unit function and weight optimization of the stochastic gradient‐based optimizer was served. The L2 penalty parameter was considered as 0.0001. A DT approach was applied, which divided data according to class purity. The RF makes a set of decision trees by bootstrap sampling from the training data. The dataset was split into a 5‐fold cross‐validation method in the approximate ratio of 4:1 to the derivation and validation sets. The four folds and one‐fold were made to train and test models, respectively. In other words, data were randomly divided into 80% and 20% for the training and the testing sets each time. Regularization was applied to handle the accuracy difference between train and test datasets, which means the model, may not generalize as well for the test set as the training set. The regularization terms were used as follows: including L2 penalty parameter for NN, Ridge L2 (standard regularizer) for stochastic gradient descent, and logistic regression. Also, for reducing the overfitting of models, the grid‐search method was applied when tuning hyperparameters and trying to select the best combination of parameters for the data. In addition, feature selection was performed to reduce the features and avoid overfitting. The flowcharts of data preprocessing and model selection are shown in Figure 1.

### 2.2 Statistical analysis

Descriptive statistics were reported as mean ± SD for continuous variables and frequency and percentage for categorical ones. The relationship between categorical variables was explained using the Chi‐square test, and the quantitative variable was tested using the independent sample t‐test. The significance level of 0.05 was considered in the analysis. The missing data were imputed using model‐based imputer methods. In this way, a separate model is constructed for each attribute. The default model is 1‐Nearest neighbor learner, which takes the value from the most similar example. Lifestyle and occupational risk factors for bladder cancer were explored using the ML approaches. The calibration plot, the area under curve (AUC) of the receiver operating characteristic (ROC) curve, precision, sensitivity, specificity, and F1 indexes were obtained to determine model performance. In the visualization, the best model was selected to calculate the probability of bladder cancer using lifestyle and occupational risk factors. All ML strategies were implemented using Orange software version 3.21.0, which builds data analysis workflows visually. The visual workflows in Orange and the dataset supporting this study's findings are available from the corresponding author upon request.

## 3 RESULTS

A total number of 1384 subjects, including 692 bladder cancer patients and 692 healthy people, were included in the study. The basic information and characteristics of the samples have been summarized in Table 1. Patients in two groups were matched based on age, BMI, and sex. In past medical history, all included chronic conditions had a significant association with bladder cancer (*p*‐value<.05). Occupational factors did not present a significant association in univariable analysis. In the case of lifestyle items, smoking, opium, and analgesics were significant factors for bladder cancer (*p*‐value<.05). Finally, fruit and vegetable use and pickles had a significant effect on bladder cancer (*p*‐value<.05). Histograms for all numerical features were presented in Supplementary 1.

**TABLE 1:** Characteristics of bladder cancer patients and healthy participants.

Variables			Group				*P*‐value
			Healthy people *N* = 692		Bladder cancer patients *N* = 692		
demographical characteristics	Age; Mean (SD)		64.92 (12.09)		65.69 (11.95)		.232
	BMI; Mean (SD)		24.81 (3.81)		24.70 (4.23)		.999
	sex	Female	129	18.6%	133	19.2%	.784
		Male	563	81.4%	559	80.8%	
	Marriage status	Married	614	88.7%	590	85.3%	.055
		Not married	78	11.3%	102	14.7%	
Past medical history	Chronic renal failure	No	656	94.8%	625	90.3%	.001
		Yes	36	5.2%	67	9.7%	
	Bladder stone	No	657	94.9%	560	80.9%	<.001
		Yes	35	5.1%	132	19.1%	
	Neurogenic bladder	No	658	95.1%	559	80.8%	<.001
		Yes	34	4.9%	133	19.2%	
	Spinal cord paralysis	No	691	99.9%	677	97.8%	<.001
		Yes	1	0.1%	15	2.2%	
	Recurrent urinary tract infection	No	631	91.2%	456	65.9%	<.001
		Yes	61	8.8%	236	34.1%	
	Diabetes mellitus	No	601	86.8%	571	82.5%	.025
		Yes	91	13.2%	121	17.5%	
	Family history of bladder cancer	No	635	91.8%	605	87.4%	.008
		Yes	57	8.2%	87	12.6%	
Occupational factors	Petroleum exposure	Not‐exposed	643	92.9%	650	93.9%	.488
		Exposed	49	7.1%	42	6.1%	
	Paint exposure	Not‐exposed	672	97.1%	667	96.4%	.449
		Exposed	20	2.9%	25	3.6%	
	Weavers and spinners jobs	Not‐exposed	672	97.1%	665	96.1%	.299
		Exposed	20	2.9%	27	3.9%	
	Leather exposure	Not‐exposed	686	99.1%	685	99.0%	.781
		Exposed	6	0.9%	7	1.0%	
Lifestyle factors	Ever smoking	Never smoker	396	57.2%	273	39.5%	<.001
		Ever smoking	296	42.8%	419	60.5%	
	Family smoke	No	587	84.8%	565	81.6%	.113
		Yes	105	15.2%	127	18.4%	
	Job smoke	No	499	72.1%	489	70.7%	.552
		Yes	193	27.9%	203	29.3%	
	Ever opium	Never used	640	92.5%	551	79.6%	<.001
		Ever used	52	7.5%	141	20.4%	
	Analgesic	Yes	161	23.3%	211	30.5%	.002
		No	531	76.7%	481	69.5%	
	Herbal drug	No	539	77.9%	524	75.7%	.339
		Yes	153	22.1%	168	24.3%	
	Hair color	not used	611	88.3%	588	85.0%	.069
		used	81	11.7%	104	15.0%	
dietary factors	Sausage usage per week	.00	556	80.3%	541	78.2%	.234
		1.00	107	15.5%	106	15.3%	
		2.00	26	3.8%	28	4.0%	
		3.00	3	0.4%	17	2.5%	
	Ham usage per week	.00	567	81.9%	550	79.5%	.341
		1.00	80	11.6%	102	14.7%	
		2.00	13	1.9%	26	3.8%	
		3.00	32	4.6%	14	2.0%	
	Can usage per week	.00	536	77.5%	537	77.6%	.906
		1.00	98	14.2%	85	12.3%	
		2.00	28	4.0%	33	4.8%	
		3.00	30	4.3%	37	5.3%	
	Fruit and Vegetables intake per day	.00	101	14.6%	124	17.9%	.003
		1.00	392	56.6%	415	60.0%	
		2.00	194	28.1%	151	21.8%	
		3.00	5	0.7%	2	0.3%	
	Pickles per day	.00	459	66.3%	495	71.5%	.032
		1.00	208	30.1%	179	25.9%	
		2.00	25	3.6%	18	2.6%	

Subsequently, various ML algorithms were applied to assess the models for predicting bladder cancer, considering lifestyle and occupational risk factors. Table 2 indicates the performances of different ML algorithms in terms of AUC, F1, precision, sensitivity, and specificity in test and train datasets, using 5‐fold cross‐validation. Considering evaluation indexes in both datasets, the RF had the preferable performance, and the RT was in the next rank. Other approaches, however, have relatively acceptable performance.

**TABLE 2:** The performance of machine learning methods in prediction of bladder cancer, in train and test datasets by 5‐fold cross validation strategy.

Approach	Dataset	Sensitivity	Specificity	Precision	AUC	F1
Neural network	Train	62.3	80.7	.75	.74	.68
	Test	59.0	75.5	.70	.71	.64
Random forest	Train	71.8	81.7	.79	.86	.75
	Test	67.2	71.9	.70	.74	.68
Decision tree	Train	67.5	75.0	.73	.78	.70
	Test	59.7	71.9	.68	.69	.63
Naive bayes	Train	59.4	78.5	.69	.73	.64
	Test	65.7	78.4	.72	.77	.71
Logistic regression	Train	60.8	78.0	.70	.74	.65
	Test	65.0	76.9	.71	.77	.68
Gradient boosting	Train	60.8	77.9	.73	.69	.66
	Test	52.0	80.6	.68	.61	.59

**FIGURE 2:**
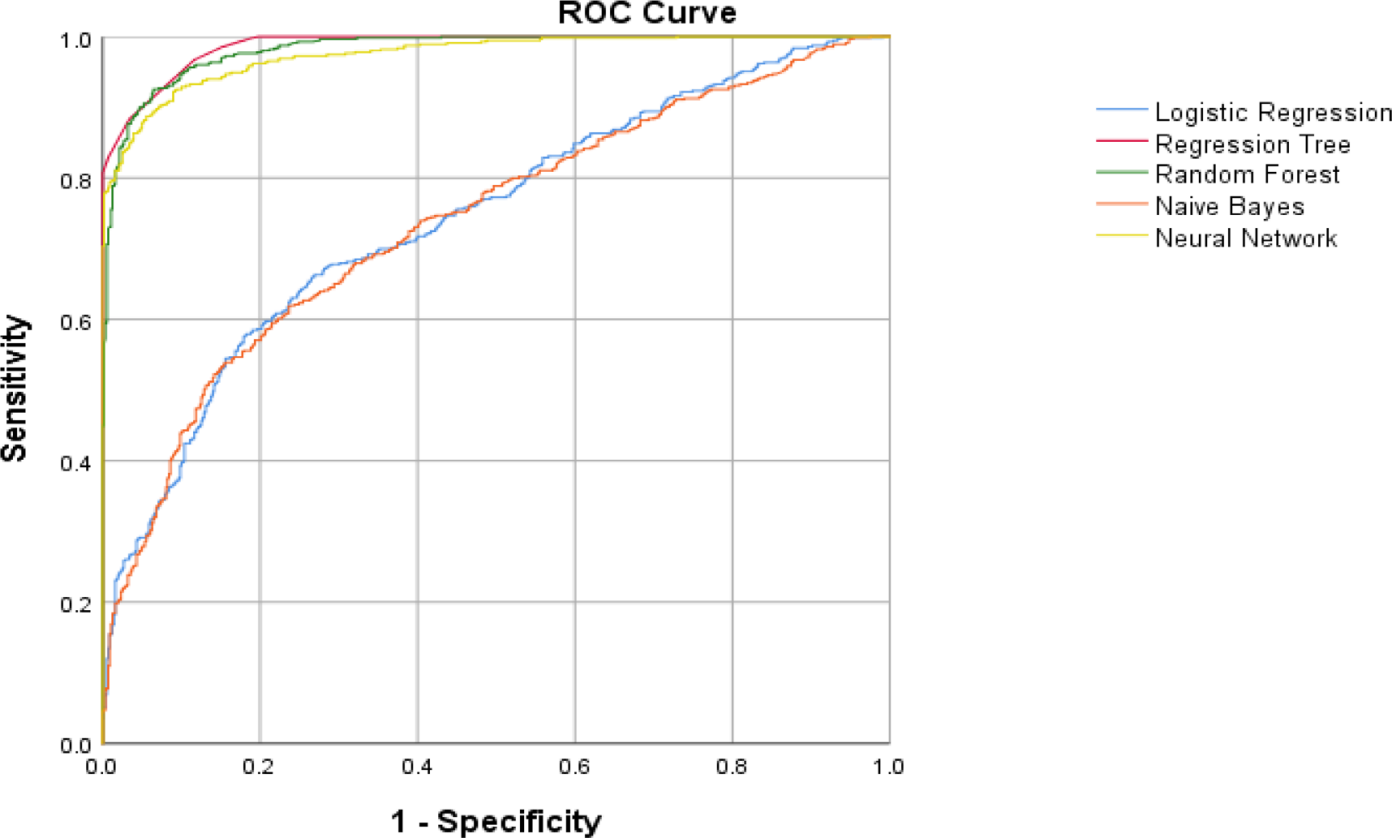
ROC curves of different ML algorithms.

**FIGURE 3:**
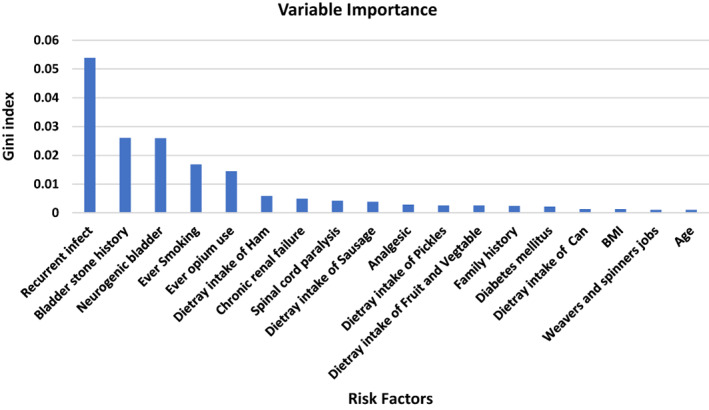
The variable importance for prediction of bladder cancer risk.

The ROC curves in Figure 2 are presented for determining the diagnostic ability of the ML algorithms. The variable importance of the RF method is plotted in Figure 3. It shows that recurrent infection, bladder stone history, neurogenic bladder, smoking and opium use, high dietary intake of ham, chronic renal failure, spinal cord paralysis, diet intake of sausage, analgesic, high dietary intake of pickles, low dietary intake of fruit and vegetable, family history of bladder cancer, diabetic mellitus, and high dietary intake of can be the most significant variables, which affect the probability of bladder cancer.

**FIGURE 4:**
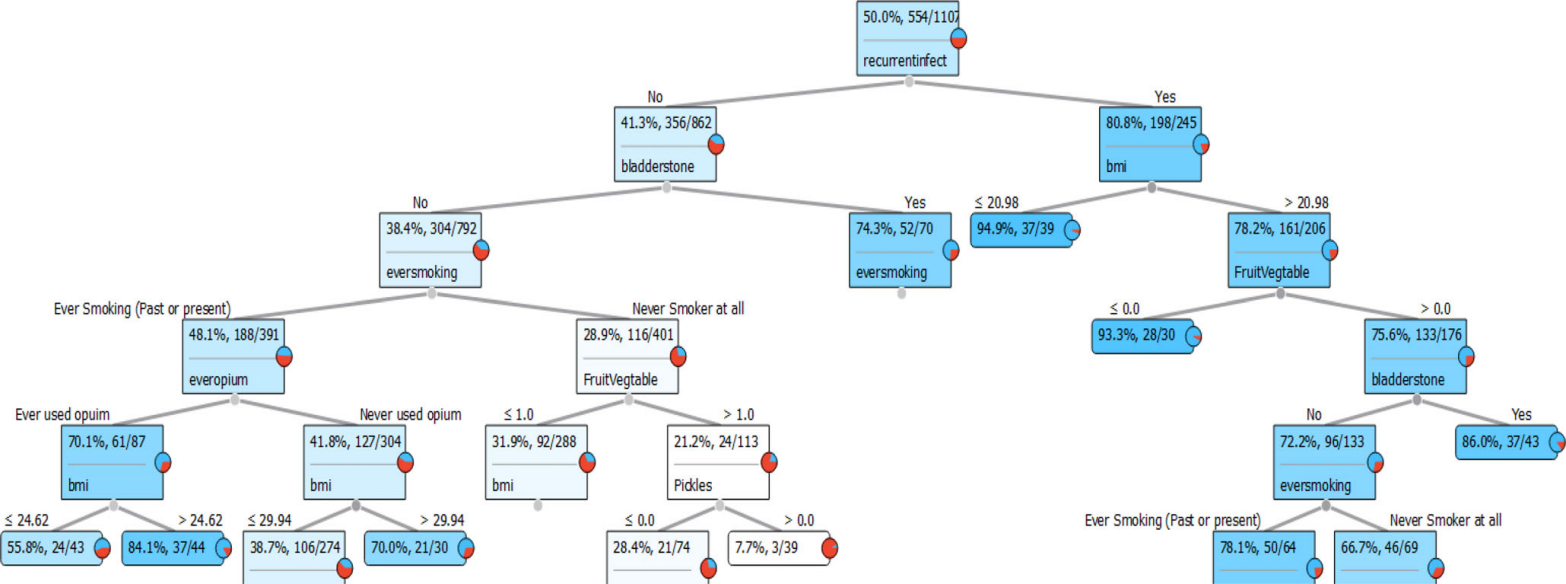
The plot displays One of the best trees in random forest to calculate the probability of bladder cancer after entering the risk factors information for a person. The maximum depth of tree was limited to 6.

Finally, a visualization of one of the best trees in random forest has been illustrated in Figure 4. To predict the outcome, start from the root node, then go to the next intermediate nodes and the edges show which subsets are looked at. One would start with the root node, which is the recurrent urinary tract infection variable, and then proceed to the next intermediate nodes and examine the edges to determine which subsets satisfy certain conditions. When the algorithm reaches the final subsets, also called leaf nodes, it evaluates the predicted outcome using the information provided in those leaf nodes. The maximum tree depths were limited to 6.

## 4 DISCUSSION

In this case–control study, we used ML methods to develop a risk prediction model for bladder cancer according to lifestyle and occupational risk factors. The univariable results presented that half of the 12 important factors of bladder cancer were related to past medical history (diabetes mellitus, chronic renal failure, bladder stone, neurogenic bladder, spinal cord paralysis, and recurrent UTI) and family history of bladder cancer. Finally, three factors depend on bladder cancer; the lifestyle items: smoking, opium, and analgesics use. The last two ones were fruits and vegetables and pickles consumption per day, which belong to the dietary factors category. In addition to the importance of acquiring a complete patient history, lifestyle, and dietary risk factors must be considered. Having a better understanding of these risk factors will prove to be a great asset to the prevention and management of bladder cancer in the future.

In 2018, a systematic review study divided the risk factors into six major groups: smoking, occupational exposure, dietary factors, environmental carcinogens, gender, race, and socioeconomic status.^9^ A more recent study in 2020 listed nine groups of risk factors: gender, age, hereditary factors, smoking, environmental and occupational exposure, alcohol, red meat, obesity, and pathogens.^10^ In our study, we found out that medical histories, such as recurrent UTI, smoking and opium use, and also daily use of vegetables, fruits, and pickles may be important factors related to bladder cancer. This is consistent with the aforementioned study; moreover, our study sheds light on the importance of past medical records of patients and emphasizes the role of smoking and dietary factors on bladder cancer. Despite their importance, a survey showed that most bladder cancer survivors were not aware of any risk factors contributing to their disease.^11^ It can be deduced that general knowledge about bladder cancer risk factors is still scarce, and we need more and more educational programs informing the audience about risk factors and how to prevent cancers. According to our results, smoking was one of the significant risk factors for patients with bladder cancer. Smoking and the use of tobacco‐related products have been the center of attention and have been considered the most significant and well‐known risk factors for bladder cancer.^9^, ^10^, ^12^

Dietary factors contain numerous variables that may lead to or prevent bladder cancer depending on their quantity and quality. In our study, the daily consumption of fruits and vegetables, and pickles was also associated with a significant reduction in the risk of bladder cancer, with an importance of fifth position in Figure 2. Studies that investigated the association between dietary factors and bladder cancer have yielded inconsistent and controversial results. According to a survey, the role of fruits, vegetables, and micronutrients is still being debated.^13^ A meta‐analysis indicated that there was no association between the risk of bladder cancer and the total amount of fluids consumed.^14^ Two dose–response meta‐analysis studies regarding tea consumption and alcohol consumption revealed no significant association.^15^, ^16^

Finally, the past medical conditions of patients must be considered when estimating the risk of bladder cancer. According to the results, we discovered recurrent UTI, neurogenic bladder, and bladder stones were all significantly associated with bladder cancer in Table 1 and had an important position in Figure 3, respectively. Metabolic syndrome, diabetes mellitus, UTIs, and parasitic infection, especially schistosomiasis, are all linked with an elevated risk of bladder cancer.^17^, ^18^

In recent years, with the development of technology, studies have used deep learning (DL) and ML models to come up with programs that can help in the diagnosis of cancers, predict their prognosis and survival outcomes, and selection the best route of treatment for patients.^19^ Considering DL and ML studies on bladder cancer, Tsai et al. predicted the neoplasm by utilizing laboratory data and ML methods. Their light GBM model differentiated bladder cancer from cystitis and other cancers with an accuracy of 84.8% to 86.9%, a sensitivity of 84% to 87.8%, a specificity of 82.9% to 86.7%, and an AUC of 0.88 to 0.92.^20^ In another study, ML algorithms were used for distinguishing bladder cancer from cystitis and predicting the survival rates of patients with bladder cancer.^21^ In a review study, Suarez‐Ibarrola et al. applied ML and DL methods for bladder cancer to predict treatment response, recurrence of tumors, and survival rates among the patients.^22^ There have not yet been ML or DP algorithms to predict the risk of bladder cancer based on risk factors in lifestyle, dietary intake, environmental, and occupational groups, and that is the novelty of our study.

The study has some strengths and limitations. We used a relatively large sample size of patients randomly chosen from the Iranian Cancer Registry system and developed risk prediction models. One limitation of our study was that we could not evaluate the effect of other risk factors. Another limitation is that dietary and lifestyle factors are greatly influenced by culture, ethnicity, and geographical and historical factors, and our study was conducted on the Iranian population, so the results might differ in various areas.

## 5 CONCLUSION

Machine learning approaches can predict the probability of bladder cancer according to medical history, occupational risk factors, and dietary and demographical characteristics.

## AUTHOR CONTRIBUTIONS

*Conceptualization*, N.B., H.A.; *Acquisition of Data*, N.S. and M.T.; *Methodology, analysis*, N.B., A.T., H.A.; *Writing‐original draft preparation*, K.S, M.P., M.T. and N.B.; *Writing‐review and editing*, N.B., A.T., H.A., and N.S.; *Administrative and Supervision*, N.B. H.A.

## CONFLICT OF INTEREST STATEMENT

The authors declare no conflicts of interest.

## ETHICAL STATEMENT

The study protocol was approved by the Ethics Committee of the Urology and Nephrology Research Center at Shahid Beheshti University of Medical Sciences. Informed consent was obtained from all subjects, and all methods were performed in accordance with the Declaration of Helsinki.

## Supporting information


**Supplementary 1.** Histograms for numerical features.

## Data Availability

Data and materials can be made available upon request.
