# Supplementary figures and images for: Lifestyle and occupational risks assessment of bladder cancer using machine learning‐based prediction models
Source: Cancer Rep (Hoboken). 2023 Jul 5;6(9):e1860. doi: 10.1002/cnr2.1860 (PMC10480417; doi:10.1002/cnr2.1860)

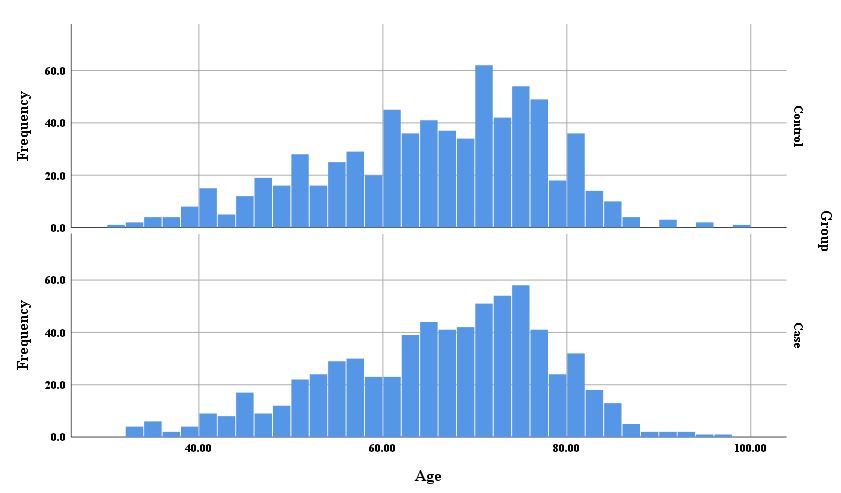

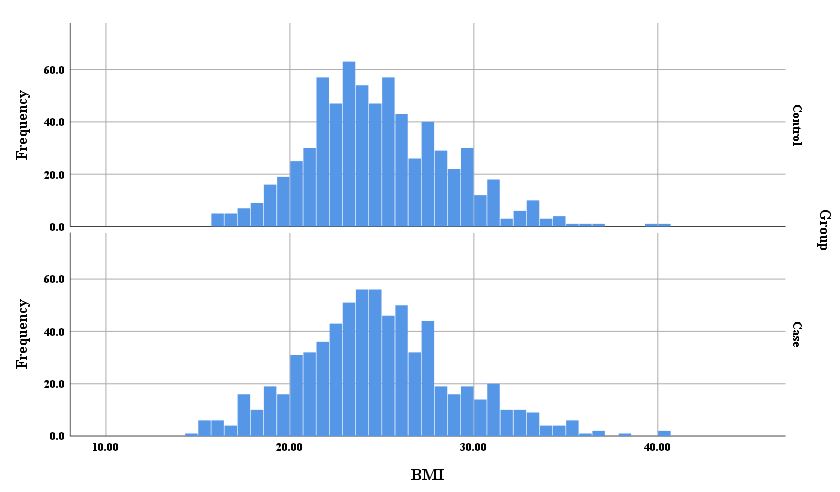


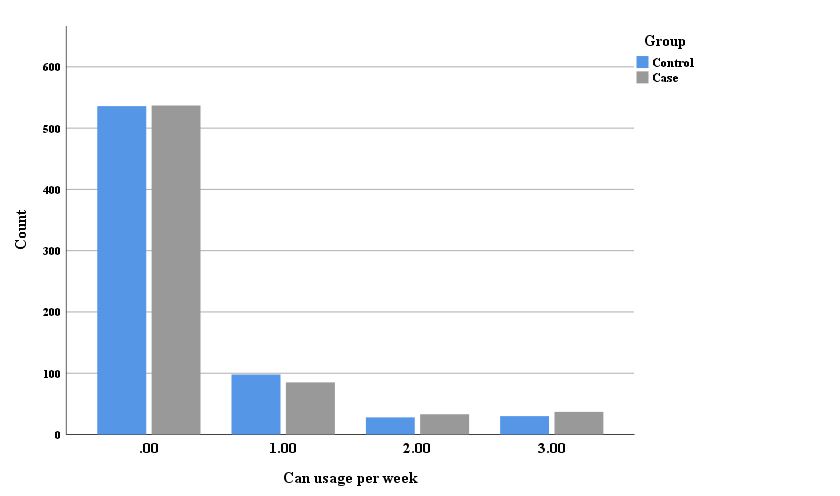

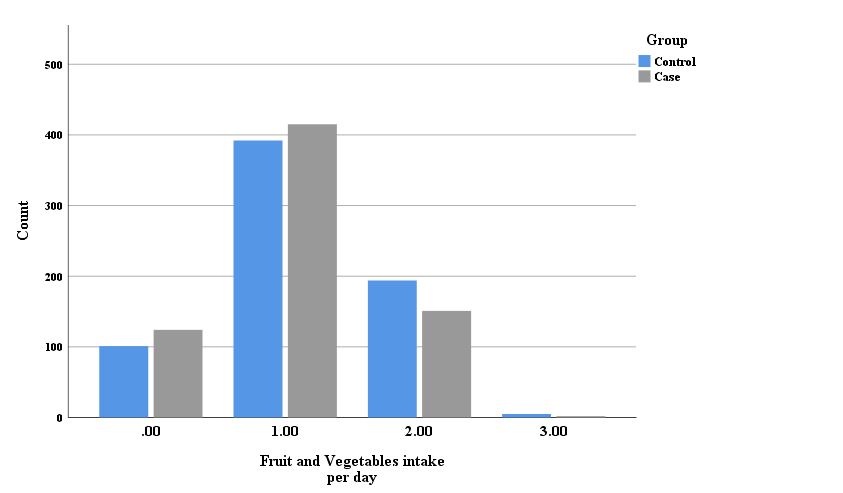

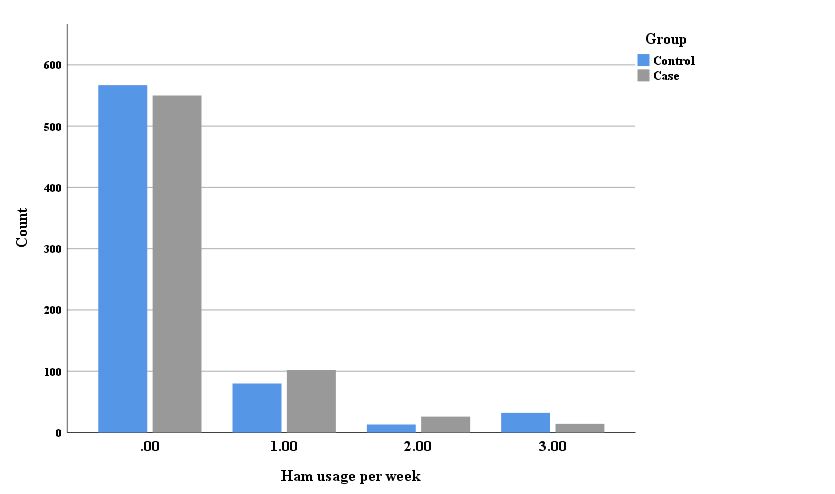

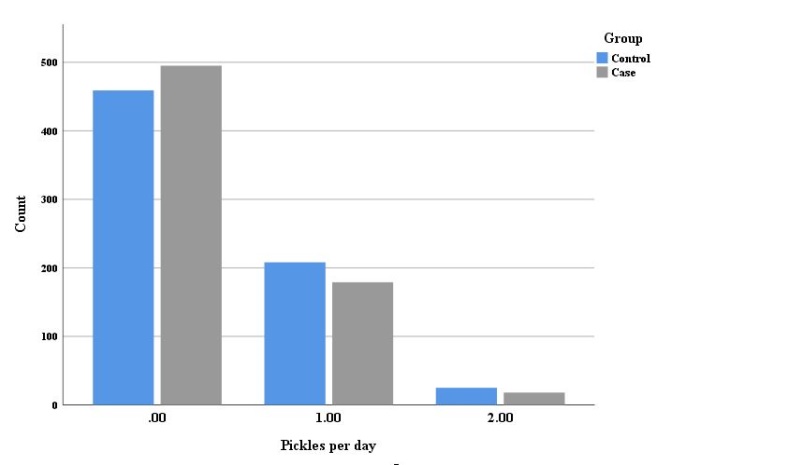

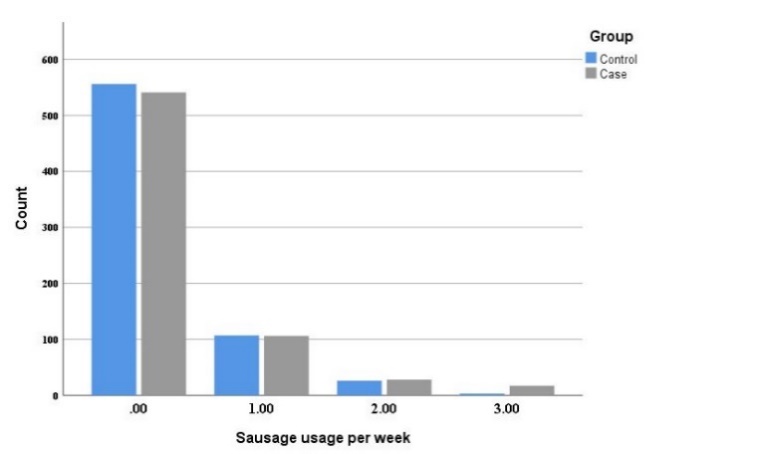
 **Supplementary 1.** Histograms for numerical features.

Supplement: Supplementary file 1 — Supplementary 1. Histograms for numerical features. [file CNR2-6-e1860-s001.docx]
